# Acquired predator recognition via epidermal alarm cues but not dietary alarm cues by isolated pupfish

**DOI:** 10.1098/rsos.230444

**Published:** 2023-09-13

**Authors:** Brian D. Wisenden, Cody M. Anderson, Kathryn A. Hanson, Molly I. M. Johnson, Craig A. Stockwell

**Affiliations:** ^1^ Biosciences Department, Minnesota State University Moorhead, Moorhead, MN 56563, USA; ^2^ Environmental & Conservation Sciences Graduate Program, North Dakota State University, Fargo, ND 58108, USA; ^3^ Biological Sciences Department, North Dakota State University, Fargo, ND 58108, USA

**Keywords:** predator recognition learning, pupfish, chemical cues, predator–prey, conservation

## Abstract

We tested whether Shoshone pupfish *Cyprinodon nevadensis shoshone* and Amargosa River pupfish *C. n. amargosae* respond behaviourally to conspecific chemical alarm cues released when epidermal tissue is damaged by a predator. We found that both subspecies reduced activity and vertical position in the water column in response to alarm cues. We then tested if pupfish can use alarm cue to acquire recognition of a novel predator. We trained pupfish with (1) water + odour of largemouth bass fed a diet of earthworms, (2) alarm cues from skin extract (epidermal alarm cues) + odour of bass fed a diet of earthworms, or (3) water + odour of bass fed a diet of pupfish (dietary alarm cues). Pupfish responded to epidermal alarm cues but not to dietary alarm cues. Pupfish were retested with the odour of bass that were fed an earthworm diet. Pupfish that had previously received epidermal alarm cues reduced vertical position and activity relative to the other two treatments. This is the first demonstration of acquired recognition of a novel predator by a pupfish, the first report of partial predator naiveté, and opens the possibility of predator-recognition training as a tool for management and conservation of endangered desert fishes.

## 1. Introduction

Costly anti-predator responses are expected to be partially or completely lost when populations experience prolonged periods of relaxed predation pressure, a condition known as predator naiveté [1,2]. Evolutionary loss of anti-predator responses is especially prevalent in small, isolated populations that occur on islands in the literal sense, or ‘islands’ of fragmented habitats that occur most commonly in freshwater aquatic habitats [3,4]. Desert fishes in the southwest of the United States represent a good example of this phenomenon. The large lakes that once covered this region disappeared after the Pleistocene (10 000 years ago) relegating surviving ichthyofauna to small, simple communities in isolated spring systems with few or no piscivorous fish predators [5,6]. Predator naiveté has been invoked to explain a decades-long pattern of extirpations and extinctions of insular fish populations in the southwestern deserts of the United States by non-native predators [7–9].

To better understand the effects of introduced predators and the potential for native desert fish species to co-persist with introduced predators, a better understanding of their anti-predator competence is needed. Given widespread use of semiochemicals to mediate predator–prey interactions [10], behavioural responses to chemical cues provide a convenient framework for investigating predator naiveté in desert fishes. Epidermal tissues damaged when a predator attacks its prey release alarm cues that are used by conspecific prey as a reliable indicator of imminent danger [10]. Prey can also detect kairomones, i.e. the body odour of predators, and dietary alarm cues, which are components or metabolites of epidermal alarm cue released from a predator's gut [11–16].

Recently, we reported a case of predator naiveté to conspecific olfactory cues in the Pahrump poolfish (*Empetrichthys latos*) [17]. This endangered species, which has been severely impacted by non-native predators [18–20], showed no behavioural response to conspecific epidermal alarm cues [17]. This is a unique case of predator naiveté because Pahrump poolfish lack the olfactoral ability to detect and avoid predation events. The poolfish study was the first reported case study linking the documented vulnerability of a desert fish to predator naiveté, motivating additional work to examine anti-predator behaviour in other endemic desert fishes. Such work is especially pressing because interactions between native prey and introduced predators continue to increase during the Anthropocene [21].

Pupfishes (superorder Acanthopterygii, order Cyprinodontiformes, *Cyprinodon* spp.) are small freshwater fishes distributed across the southwestern region of the United States, northern Mexico, and extend eastward mainly along coastal waters as far north as New York, and extend southward into the Caribbean Island archipelago [22]. Throughout the southwest region of the United States, desiccation of the Pleistocene Lakes left various pupfish species and populations isolated in remnant springs and low-gradient streams [23]. Consequently, pupfish species in this region have small geographical ranges and high levels of endemism. They typically occur in simple communities often lacking large piscivorous fish [17]. Pupfish have a mixed success in cohabiting with non-native species. Non-native largemouth bass *Micropterus salmoides* played a key role in the extinction of the last natural population of the Santa Cruz pupfish *Cyprinodon arcuatus* [8,9,24]. However, non-native mosquitofish *Gambusia affinis*, that prey on larval Mohave tui chub *Siphateles bicolor mohavensis* [25] and larval Pahrump poolfish *Empetrichthys latos* [18,19], have co-persisted with pupfish for decades [18,26].

The ability to adapt and co-exist with non-native species is likely explained by complex ecological and behavioural interactions over ecological and evolutionary time [27]. Predator naiveté occurs when prey fail to recognize a predator as a threat, recognize the predator as a threat but fail to execute a response that is effective, or recognize and respond, but are overwhelmed by a super predator beyond the ecological or evolutionary experience of the prey species [28]. Further, predator naiveté may operate at multiple levels [27–29]. For example, prey can learn to recognize cues of non-native predators if they retain robust mechanisms of recognizing general traits of predators such as a carnivorous diet, forward-facing eyes, body size and movement patterns [27]. As described below, many fishes acquire predator recognition through associative learning, thus do not rely on genetic templates for predator recognition. On the other hand, if some forms of labelling are lost due to long periods of isolation from a class of predators, then prey may experience partial naiveté and lack the ability to recognize a non-native predator [27].

The first experiment in the present study set out to test if naiveté to conspecific alarm cues previously observed in Pahrump poolfish [17] generalizes to other desert fishes. We experimentally tested for behavioural responses to conspecific alarm cues for two isolated pupfish subspecies, Shoshone pupfish *Cyprinodon nevadensis shoshone* and Amargosa River pupfish *C. n. amargosae*. Similar to the Pahrump poolfish, these pupfish subspecies have been long isolated in the Death Valley region of California, USA [30]. *C. n. shoshone* was thought extinct until it was rediscovered in 1986 [31]. Shoshone pupfish evolved in allopatry as the only fish species within its biological community, whereas Amargosa River pupfish evolved in sympatry with Amargosa Canyon speckled dace *Rhinichthyes osculus nevadensis* [30,32] and have co-persisted with non-native western mosquitofish *Gambusia affinis* for several decades. These two pupfish subspecies qualify as good candidates to test the effect of isolation on predator naiveté, or at least, naiveté to conspecific alarm cue.

The second experiment presented here built on the findings from the first experiment. If pupfish detect and respond to conspecific alarm cue, then these cues may also facilitate associative learning of predator identity [10,27,33]. Alarm cue, i.e. either those derived from damaged skin (epidermal alarm cue) or those released from the gut of a predator (dietary alarm cue), may also serve to facilitate associative learning [11,12,14,15,33–38]. Recognition learning allows fish to acquire recognition of correlates of risk (such as a predator's odour or appearance) that give prey a mechanism for early detection of risk in future encounters [35]. A single pairing of alarm cue and a novel stimulus is sufficient to form an association between the novel stimulus and predation risk [10,33]. This mode of learning has been demonstrated for many species to train fish to fear visual [39], olfactory [34,36] and auditory [40] correlates of predation. Such recognition learning can be used as a management tool to train captive-reared animals to recognize predators before they are released into the wild [41,42].

## 2. Material and methods

Shoshone pupfish were collected from two sites in the Shoshone wetlands near Shoshone, California in May 2021, using standard Gee minnow traps. Amargosa River pupfish were also captured in May 2021 using minnow traps in the Amargosa River near Tecopa, California. Fish were transported to Minnesota State University Moorhead and held in 378 l population-specific holding tanks filled with dechlorinated tap water (approx. 0 ppt salinity), maintained on a 12 : 12 D : L cycle at 24°C and fed a diet of freshly thawed brine shrimp (*Artemia sp.*) and commercial flake food (TetraMin Tropical Flakes, Blacksburg, VA).

### 2.1. Experiment 1: behavioural responses to conspecific epidermal alarm cue

#### 2.1.1. Cue preparation

Chemical alarm cues were prepared following the protocol provided by Wisenden [43]. Donor fish were euthanized with an overdose of MS-222 (tricaine methanesulfonate) and cervical dislocation before the epidermis of each fish was removed (NDSU IACUC Protocols A18054 and A21042, MSUM IACUC Protocol 19-R/T-BIO-018-N-Y-C). Skin fillets were removed from each side of the fish and measured to calculate total skin area, then placed in a beaker of dechlorinated tap water resting on a bed of crushed ice. Skin fillets were homogenized with a handheld blender for 3 min, filtered through a loose wad of polyester fibre, and diluted with dechlorinated tap water to a final concentration of 1 cm^2^ of skin per 10 ml. Chemical alarm cue was then aliquoted into 10 ml doses and stored at −20°C until needed. We prepared separate epidermal alarm cue from Shoshone pupfish and Amargosa River pupfish. Blank dechlorinated tap water controls of 10 ml each were prepared and frozen at −20°C until needed.

#### 2.1.2. Experimental protocol

Trials were conducted by testing single focal fish in 37 l glass aquaria with a 5 cm × 5 cm grid drawn on the short side of the tank. All fish were females to avoid introducing variance attributable to sex differences in activity (e.g. [44]). Opaque dividers were placed between adjacent aquaria to visually isolate focal fish. An air stone supplied oxygen to the tanks and a separate stimulus delivery tube secured to the air stone was used to deliver test stimuli into the tank. Focal fish were acclimated for a minimum of 20 h before being tested. Each fish was fed at least 20 min before the start of the trial to reduce overall stress.

All observations were recorded using a Canon VIZIA HF R700 video camera positioned in front of the test tank. We recorded activity and vertical position in the water column because these measures are common components of anti-predator behavioural responses that significantly reduce predation risk [10,43]. Predators locate prey by detecting motion, therefore inactivity, especially within structure on the substratum, is a general response to threat of predation. Activity was measured by counting the total number of lines crossed by the fish's eye during 5-min pre- and 5-min post-stimulus observation periods. Vertical position was recorded every 10 s for both pre- and post-stimulus periods by noting the horizontal row in the grid occupied by the test subject (1 = tank bottom, 5 = water surface).

Each species of pupfish was tested with either conspecific alarm cue or dechlorinated tap water (control). Each fish was tested only once. Both Amargosa River pupfish and Shoshone pupfish were tested on the same days, i.e. a block of four trials, one of each treatment type: Amarogosa-Alarm, Amargosa-Water, Shoshone-Alarm, Shoshone-Water, to control for any unmeasured extraneous influences on fish response. Fish that did not exhibit normal behaviour and were not consistently active in the pre-stimulus observation period were excluded from analysis because they would not provide a valid assessment of the effect of injected test stimuli. Thus, we excluded all trials where fish did not move within at least one of the five 1-min intervals during the pre-stimulus observation period. Deletion of inactive fish removed one trial from Amargosa-Alarm, six trials from Amargosa-Water, but none of the trials using Shoshone pupfish. Final samples sizes were Amargosa-Alarm (*n* = 24), Amargosa-Water (*n* = 19), Shoshone-Alarm (*n* = 25), Shoshone-Water (*n* = 27).

#### 2.1.3. Statistical analysis

Activity (total number of lines crossed in 5 min) and Vertical Position (average grid row in the water column) were analysed via analysis of covariance (ANCOVA) using Cue as a categorical predictor and Pre-stimulus behaviour as a covariate. The initial analyses included ‘Block’, but this term was dropped from final models because it was not significant. A significant effect of cue was indicated by a significant (*p* < 0.05) main effect of Cue, or a significant effect of the interaction between Cue and Pre-stimulus behaviour, i.e. that the relationship between Post-stimulus behaviour and Pre-stimlulus behaviour depends on the type of Cue introduced. Software used for analysis was JMP Pro 15.

### 2.2. Experiment 2: acquired predator-recognition learning in Shoshone pupfish using epidermal and dietary alarm cue

#### 2.2.1. Preparation of test cues

Epidermal alarm cues were prepared as described above from four pupfish (2M, 2F) euthanized by an overdose of MS222 (methanetricaine sulfonate). We harvested a total of 26.92 cm^2^ of skin fillets. Skin fillets were homogenized with a hand blender, filtered through a loose wad of polyester fibre and diluted with deionized water to produce 20, individual 10 ml doses, each containing the equivalent of about 1.5 cm^2^ of skin. These were frozen at −20°C until needed. Blank water control cue was prepared by freezing 10 ml doses of deionized water.

Predator odour was collected following previously established protocols [11,13,16,36]. Largemouth bass *Micropterus salmoides* (mean ± SE TL = 110.8 ± 4.2 mm, *n* = 5) were collected by seine net from Silver Lake, MN. Predator odour was prepared from five bass fed a diet of earthworms (*Lumbricus terrestris*) to control for diet effects [11,36]. Bass were placed together in a tank 92 cm × 46 cm filled to a depth of 15 cm (volume = 64 l) with fresh dechlorinated tap water for 30 h. Tank water was collected and stored in 60 ml aliquots and frozen at −20°C until needed. This odour of bass on a diet of earthworms is referred to as Bass Odour (E).

Dietary alarm cues were collected from the same five bass after they consumed seven Shoshone pupfish. The five bass were presented with five pupfish (3F, 2M), of which four were eaten, the fifth was removed the following day when five additional pupfish were added (3M, 2F), of which three were eaten for a total consumption of seven pupfish over two days. The five bass were then moved to a new holding tank containing 64 l of fresh dechlorinated tap water to digest for 30 h. Tank water was collected and stored in 60 ml aliquots and frozen at −20°C until needed. This odour of bass on a diet of pupfish is referred to as Bass Odour (P).

#### 2.2.2. Experimental protocol

Individual pupfish were placed in 37 l test aquaria equipped with an air stone and injection tube, and a 5 cm × 5 cm grid on the front panel of the aquarium for scoring behaviour, as described for the first experiment. In the second experiment, we tested females and males, split evenly among treatment groups, to test for an effect of sex, if any, on behavioural responses to indicators of predation risk. In addition, a mirror was placed along one side of each tank because isolated pupfish are relatively inactive but increase activity when in the company of their own reflection. This small change in methodology eliminated the problem of inactive fish during pre-stimulus periods that we encountered in the first experiment.

We recorded Vertical Position (horizontal row in the grid occupied by the fish at 10-s intervals), and Activity (number of grid lines crossed summed over 5 min), as described for the first experiment. To train pupfish to associate bass odour with predation risk, each fish was tested twice (table 1). In the first trial fish were trained with one of three treatments: (1) blank water + bass odour (E) (=water control treatment), (2) epidermal alarm cue + bass odour (E) (=epidermal alarm cue treatment), or (3) blank water + bass odour (P) (=dietary alarm cue treatment). Treatments were blocked with respect to time and spatial arrangement in the laboratory to control the effect, if any, of unmeasured variables.

**Table 1.** Experimental design. Pupfish were tested twice, once in a training trial and once in a test trial. Training trials presented combinations of water, alarm cue derived from skin extract (epidermal alarm cues), alarm cue derived from digestion of pupfish prey (dietary alarm cues, Bass Odour (P)) and bass odour maintained on a diet of earthworms (Bass Odour (E)). All fish were tested with Bass Odour (E) in test trials. *N* = 16 per treatment group.

treatment group	training trials	test trials
control	water + bass odour (E)	bass odour (E)
epidermal alarm cue	alarm cue + bass odour (E)	bass odour (E)
dietary alarm cue	dietary alarm cue + bass odour (P)	bass odour (E)

After a single training trial, tanks were drained to a depth of 3 cm and refilled with fresh dechlorinated water. Injection tubes were replaced after every trial. After 24–48 h, each fish was tested for a response to bass odour (E) (table 1). We conducted 48 trials with *n* = 16 trials for each treatment group. Within each treatment group, *n* = 8 trials were conducted on females and *n* = 8 trials were conducted on males. We did not have enough male Shoshone pupfish to complete trials on males; therefore we used male Amargosa River pupfish for five trials, spread evenly across treatment groups.

#### 2.2.3. Statistical analyses

Data for training trials were analysed separately from data from test trials. In each case, using the same approach used to analyse data in the first experiment, Activity (total number of lines crossed in 5 min) and Vertical Position (average position in water column) were analysed via analysis of covariance (ANCOVA) using Cue and Sex as categorical predictors and Pre-stimulus behaviour as a covariate. Three-way and two-way interaction terms that did not surpass the threshold of significance (*p* > 0.05) were deleted to simplify the model and to conserve degrees of freedom. Least significant difference *post hoc* tests were used for planned pairwise comparisons among epidermal alarm cue, dietary alarm cue and control cue treatments. The software used for analysis was SPSS v. 26.

## 3. Results

### 3.1. Experiment 1: behavioural responses to conspecific epidermal alarm cue

**Figure 1.**
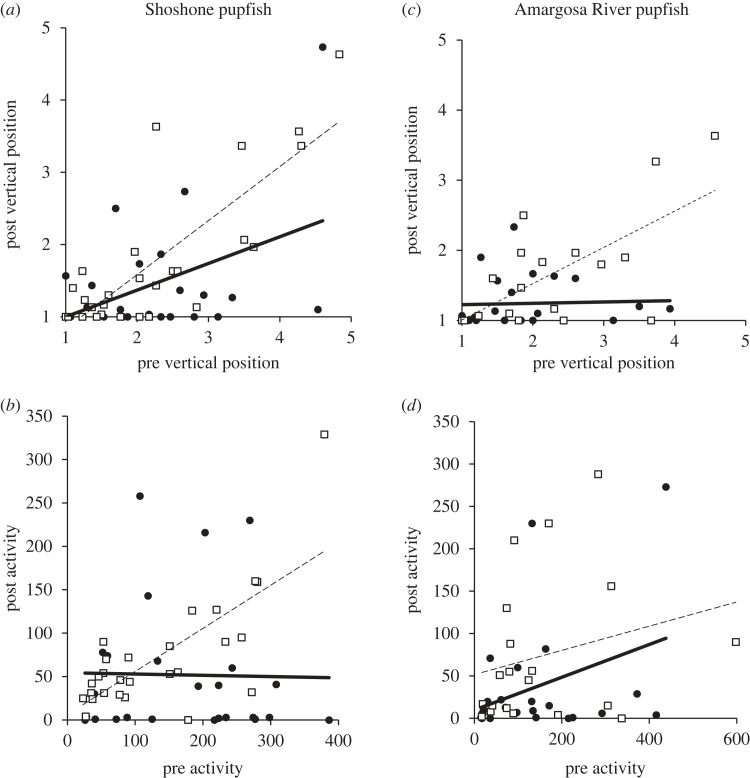
Death Valley pupfish change in behaviour in response to conspecific alarm cue (filled circles, bold lines), or water (open squares, dashed lines). (*a*) Shoshone pupfish change in Vertical Position (1 = surface, 5 = bottom); (*b*) Shoshone pupfish change in Activity (=total grid lines crossed in 5 min); (*c*) Amargosa pupfish change in vertical position; (*d*) Amargosa pupfish change in activity.

For Shoshone pupfish the interaction between pre-stimulus behaviour and cue treatment had a significant effect on post-stimulus vertical position (pre-stimulus Vertical Position × Cue: *F*_1,48_ = 4.16, *p* = 0.047; figure 1*a*) and post-stimulus activity (pre-stimulus Activity × Cue: *F*_1,48_ = 8.26, *p* = 0.006; figure 1*b*). Relative to pre-stimulus behaviour, Shoshone pupfish reduced activity and lowered their position in the water column following exposure to conspecific alarm cue. For Amargosa River pupfish, the interaction between cue type and pre-stimulus behaviour had a significant effect on vertical position (pre-stimulus Vertical Position × Cue: *F*_1,39_ = 10.49, *p* = 0.002; figure 1*c*), indicating movement toward the bottom in response to conspecific alarm cue. However, Amargosa River pupfish activity was not significantly affected by epidermal alarm cue (Cue: *F*_1,39_ = 1.82, *p* = 0.185, pre-stimulus Activity × Cue: *F*_1,39_ = 0.162, *p* = 0.689; figure 1*d*).

### 3.2. Experiment 2: acquired predator-recognition learning using epidermal and dietary alarm cues

#### 3.2.1. Training trials

**Figure 2.**
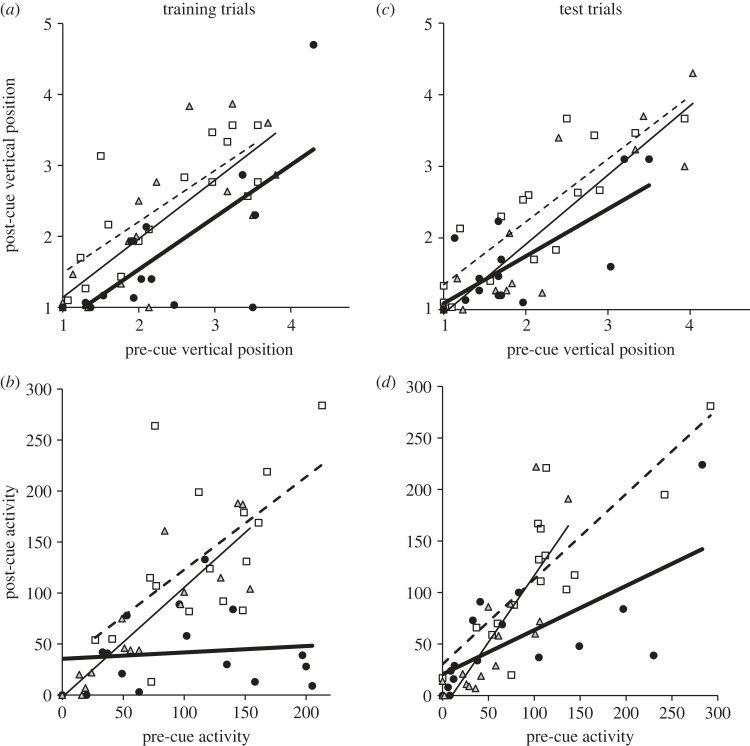
Post-cue behaviour as a function of cue treatment and pre-cue behaviour. Open squares and dashed line, water + bass odour on earthworm diet; shaded triangles and solid line, water + bass odour on pupfish diet (dietary alarm cues); filled circles and bold line, epidermal alarm cues + bass odour on earthworm diet. (*a*) Training trials for change in Vertical Position (1 = surface, 5 = bottom); (*b*) training trials for change in Activity (total grid lines crossed in 5 min); (*c*) test trials for change in Vertical Position; (*d*) test trials for change in Activity.

Cue type in training trials had a significant effect on post-stimulus vertical position (Cue: *F*_2,43_ = 4.603, *p* = 0.015; figure 2*a*) and post-stimulus activity (pre-stimulus Activity × Cue *F*_2,39_ = 10.009, *p* < 0.001; figure 2*b*). *Post hoc* pairwise comparisons revealed that pupfish reduced vertical position in response to epidermal alarm cues + bass odour (E) relative to pupfish that received water + bass odour (E) and compared to fish that received dietary alarm cues, i.e. water + bass odour (P), while the latter two treatments did not differ from each other (table 2). Similarly, *post hoc* tests showed that pupfish reduced activity in response to epidermal alarm cues + bass odour (E) relative to those that received water + bass odour (E) and those that received dietary alarm cues, i.e. water + bass odour (P), while the latter two treatments did not differ from each other (table 2).

**Table 2.** *p*-values for planned *post hoc* comparisons (least significant differences) among treatment groups for training trials and test trials. Bolded values are *p* < 0.05. Treatment groups are as described in table 1.

	vertical position	activity
training trials		
epidermal alarm cue v. control	**0.005**	**<0.001**
dietary alarm cue v. control	0.360	0.454
epidermal alarm cue v. dietary alarm cue	**0.047**	**0.001**
test trials		
epidermal alarm cue v. control	**0.016**	**0.011**
dietary alarm cue v. control	0.076	0.845
epidermal alarm cue v. dietary alarm cue	0.482	**0.027**

Females and males responded similarly in vertical position (*F*_1,43_ = 0.188, *p* = 0.667) but there was a significant interaction between sex and cue in activity (*F*_2,39_ = 3.350, *p* = 0.045) because males were less active than females in trials that received water + bass odour (E) and water + bass odour (P) but more active than females in trials that received epidermal alarm cue + bass odour (E) (data not shown).

#### 3.2.2. Test trials

Conditioning experience in training trials had a significant effect on behavioural response to bass odour (E) in test trials in terms of reduced vertical position (Cue: *F*_2,43_ = 3.333, *p* = 0.045; figure 2*c*) and reduced activity (pre-stimulus Activity × Cue: *F*_2,41_ = 6.043, *p* = 0.005; figure 2*d*). *Post hoc* pairwise comparisons (table 2) revealed that pupfish previously conditioned with epidermal alarm cues + bass odour (E) reduced vertical position relative to pupfish that were previously conditioned with water + bass odour (E) but not compared to fish previously conditioned with dietary alarm cues, i.e. water + bass odour (P). Fish previously conditioned with water + bass odour (E) did not differ in vertical position from fish previously conditioned with dietary alarm cues, i.e. water + bass odour (P). Similarly, for activity *post hoc* tests showed that pupfish that had been trained with epidermal alarm cues + bass odour (E) reduced activity in response to bass odour (E) relative to those that had previously received water + bass odour (E) and those that had received dietary alarm cues, i.e. water + bass odour (P), while the latter two treatments did not differ from each other for either (table 2). Responses tended to be more pronounced in males than females in vertical position (*F*_1,43_ = 3.196, *p* = 0.081) and activity (*F*_1,41_ = 3.637, *p* = 0.064) but these effects did not surpass statistical significance (data not shown).

## 4. Discussion

Reductions in vertical position and activity in response to conspecific alarm cue are consistent with anti-predator responses to conspecific alarm cue observed in many other fishes [10]. These findings differ from the results found for another insular desert fish, the Pahrump poolfish, which did not respond to conspecific alarm cue [17]. Pahrump poolfish, and Amargosa River pupfish and Shoshone pupfish all share a similar history of evolving with limited piscivorous predation pressure since the end of the Pleistocene; thus we hypothesized that Amargosa River and Shoshone pupfish may also lack behavioural responses to alarm cues. Alarm reactions by Amargosa pupfish presented here corroborate field reports and mesocosm experiments that showed that pupfish can co-persist with non-native predators such as crayfish [26,45]. Thus, isolation from piscivorous fish would seem to be insufficient, in and of itself, to result in evolutionary naiveté.

While pupfish had no pre-existing recognition of bass odour, pupfish were able to use epidermal alarm cues to facilitate associative learning to acquire recognition of novel predator odour. This finding suggests the possibility that pupfish could use epidermal alarm cues to acquire recognition of novel non-native predators introduced into isolated desert populations. An argument can made for selection for anti-predator behaviour in the absence of piscivorous fish because small fishes in isolated springs are exposed to predation by birds and large invertebrates that have the ability to disperse to isolated habitats [46]. Little is known about the role of predation from these sources on larval recruitment and population dynamics in desert fishes.

The second experiment independently corroborated findings from the first experiment. Both sexes of Shoshone pupfish responded to alarm cues in skin extract, which is a novel finding because the first experiment used female fish exclusively. Inconsistent sex effects in this study may be an artefact of low sample size. Additional experimental work is needed to resolve the effect of sex, if any, in risk assessment.

There was scant evidence that pupfish could detect dietary alarm cues directly, nor could they use dietary alarm cues to acquire recognition of novel predator odour via associative learning. This is an unexpected result because many species readily use dietary alarm cues for detection and learned recognition of predators (e.g. [11,13,15,16,36,47,48]). One possible explanation for why there might be a response to epidermal alarm cues but not to dietary alarm cues is partial predator naiveté [27]. Shoshone springs and associated wetlands are not predator-free. Odonate nymphs and various piscivorous bird species frequent this area and presumably prey on pupfish. Invertebrate predators such as dragonfly nymphs would cause extensive damage to the epidermis, which would release epidermal alarm cue. Thus, we hypothesize that these non-fish predators may sustain selection for a chemically mediated epidermal alarm cue system for risk detection and assessment in these pupfish. However, these isolated waters have not contained piscivorous fish species and the dietary alarm cues they release for thousands of years [6]; therefore pupfish may have lost the ability to detect and respond to dietary alarm cues. This would constitute a case of partial predator naiveté [27].

Carthey & Blumstein [27] outlined multiple paths in eco-evolutionary time for interactions between prey and predators that may appear and disappear over ecological and evolutionary time. Our data fit two of their proposed scenarios simultaneously. Clearly, recognition of epidermal alarm cues has been maintained, likely due to ongoing stabilizing selection from predation by birds and invertebrates. Recognition learning has also been conserved allowing pupfish plasticity and adaptability to respond to indicators of risk that shift dynamically over ontogenetic, ecological and evolutionary time scales (Figure 1 S7 in [27]). However, it may be the case that *C. n shoshone* has lost the ability to label fish predators through dietary alarm cues resulting in partial naiveté (Figure 1 S8 in [27]).

Responses to dietary alarm cues are common in other taxa [15]. Cladocerans, echinoderms and amphibians may ignore chemical cues released directly from damaged conspecifics but respond when those cues are combined with kairomones of, or digested by, their respective predators. For example, Stabell *et al*. [48] reported that alarm cues of water fleas *Daphnia galeata* are activated only after digestion by their predator, the three-spine stickleback *Gasterosteus aculeatus*, or biochemical breakdown of daphnia mash when mixed with digestive enzymes contained in the contents of fish intestine, or when daphnia mash is left to decompose by bacterial degradation for 12 h. Similarly, amphibian tadpoles respond with significantly greater intensity to chemical cues from crushed conspecifics that have been consumed compared to cues from crushed conspecifics that have not been consumed [49]. Finally, urchins *Strongylocentrotus droebachiensis* respond significantly more intensely to kairomone of echinovorous wolffish *Anarhichas lupus* (including dietary alarm cues) than they do to undiluted urchin extract [50].

The absence of a response to dietary alarm cues by pupfish in this study is highly unusual, and suggests that they did not recognize the metabolites of epidermal alarm cue due to predator naiveté. The method of collecting dietary alarm cues used in this study was similar to the methods used in similar studies that have shown strong responses in other taxa (e.g. [16]). It may be the case that dietary alarm cues of pupfish are particularly quick to degrade beyond recognition; however, given the general role of dietary alarm cues across many other taxa [15] our data suggest that pupfish simply did not recognize dietary alarm cues due to partial predator naiveté.

Such partial predator naiveté may explain the rapid decline of the Santa Cruz pupfish population following the introduction of largemouth bass [9]. The release of conspecific alarm cues from bass predation events may be limited due to the suction-feeding mode of prey capture that bass use to ingest small-bodied fish such as pupfish [51]. To recognize predators that employ suction feeding, prey would need to rely on secondary cues of predation risk such as dietary cues, which our data suggest is a source of information that may not be available to pupfish. Shoshone pupfish would need to rely on size classes of bass too small to swallow pupfish whole to facilitate predator recognition learning.

Differential responses to epidermal and dietary alarm cues suggest that these two forms of alarm cue may differ biochemically and rely upon independent proximate mechanisms of physiological detection and cognitive recognition. Unlike well-studied pheromone systems in arthropods and secondary compounds used by plants to deter herbivory, chemical characterization of alarm cues in fishes is in its infancy [52]. The little work that has been done on fish has focused on fishes in the superorder Ostariophysi [53,54]. Candidate compounds that may serve as one of the active ingredients of epidermal alarm cue include hypoxanthine 3N oxide [55–57], chondroitin sulfate [58,59], or other unidentified polypeptides [60,61]. Further, there is evidence that the source may be, in part, from epidermal club cells [53,54,62] (but see [63]) or resident surface bacteria [64]. Pupfish are in the superorder Acanthopterygii, possess some epidermal club cells [54], but the biochemistry of skin extract and alarm cue is unstudied in this group. Chemical characterization of dietary alarm cues has received almost no attention but what little work that has been done suggests that they are metabolites of epidermal alarm cue that survive biochemical digestive processes [48,65].

Predator recognition learning demonstrated in this study raises the potential for predator-recognition training as a tool for resource management and conservation for augmenting and/or restoring imperilled species to their former native range [27,41,42,66–69]. Predator recognition training programmes have successfully implemented and in some cases demonstrated improved post-release survival (e.g. fishes: sturgeon [70] salmonids [71–73], cichlids [74] and walleye [75]) and other vertebrates (amphibians [76]; mammals [77–81]; non-avian reptiles [82]; avian reptiles [83–88]). Further, training could be useful for cases where individuals are translocated among habitats to increase gene flow [89–91] or establish new populations, which are practices widely employed with protected fishes in the western United States [92,93].

This study provides evidence for anti-predator behavioural responses to conspecific alarm cues in isolated desert pupfishes. In a similar study, we reported anti-predator behaviours in response to conspecific alarm cues across a cline of predation for populations of Red River pupfish *Cyprinodon rubrofluviatilis* [94]. Further, Shoshone pupfish can acquire predator recognition of a novel predator through epidermal alarm cue-induced recognition learning even though they have been isolated from piscivorous fish for approximately 10 000 years. This finding indicates that pupfish have some capacity to adapt to and coexist with introduced predators, and that they may be good candidates for predator-recognition training as a management tool. Pupfish in this study failed to respond behaviourally to dietary alarm cues, possibly due to partial predator naiveté. Consequently, pupfish were not able to associate risk with a novel predator odour on the basis of dietary alarm cues. For predators such as largemouth bass that employ a suction-feeding mode of prey capture, where detectable levels of epidermal alarm cues are not released during predation, the inability to detect and respond to dietary alarm cues may increase vulnerability of pupfish to population reduction or extirpation if ever they encounter suction-feeding predators.

## Data Availability

Dryad Digital Repository: https://doi.org/10.5061/dryad.rv15dv4cc [95].
